# Supplementary material for: VO: The Vaccine Ontology
Source: bioRxiv. 2025 Aug 15:2025.08.12.669998. Preprint. [Version 1] doi: 10.1101/2025.08.12.669998 (PMC12363946; doi:10.1101/2025.08.12.669998)
Supplement: Supplement 1 [file media-1.pdf]

Supplemental Materials:

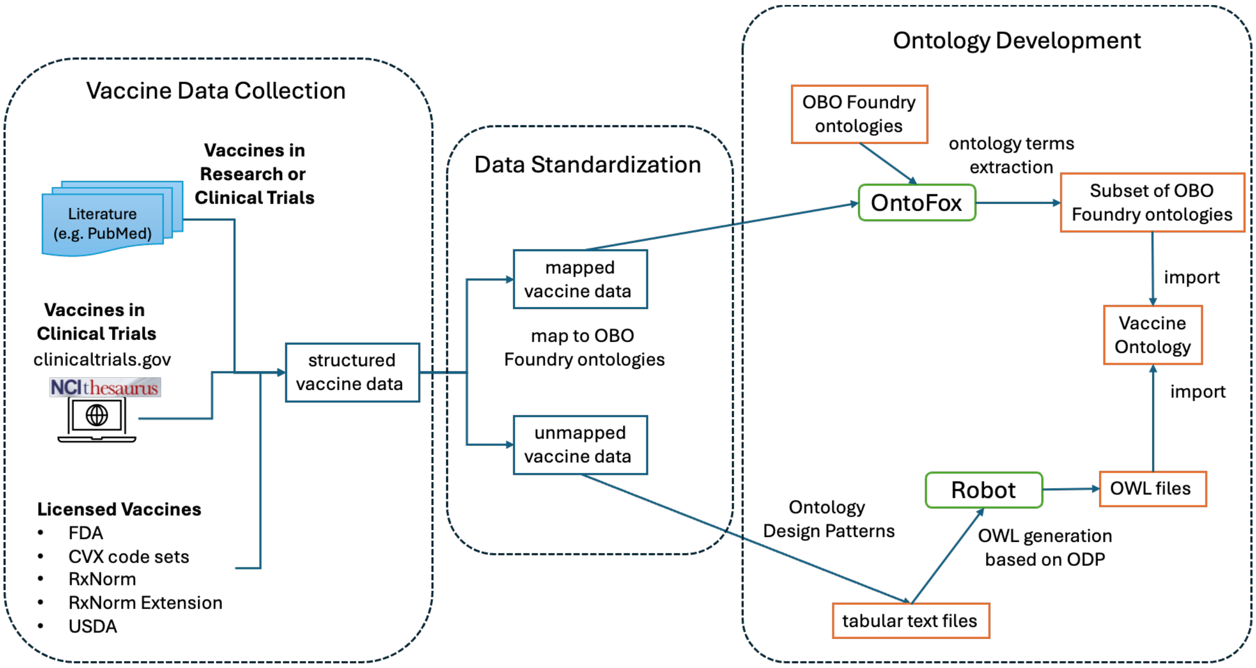

Supplemental Figure 1. Overview of VO development framework. Abbreviations: FDA: Food and Drug Administration; CVX: Centers for Disease Control and Prevention; USDA: U.S. Department of Agriculture; OBO: Open Biological and Biomedical Ontology; ODP: Ontology Design Pattern.
